# Supplementary material for: Genome-wide analysis of gibberellin-dioxygenases gene family and their responses to GA applications in maize
Source: PLoS One. 2021 May 7;16(5):e0250349. doi: 10.1371/journal.pone.0250349 (PMC8104384; doi:10.1371/journal.pone.0250349)
Supplement: S1 Table — (DOCX) [file pone.0250349.s001.docx]

**S1 Table. Primers used in present study.**

| Gene | Sequence (5'-3') | Product (bp) |
| --- | --- | --- |
| *ZmGA2ox1* | Forward: AGAGACTTCACCTTCGGGGA | 99 |
|  | Reverse: TGGCCGGTGTTTGAGAAAGT |  |
| *ZmGA2ox4* | Forward: GAGGGAGAGGAGAGCCTGTA | 112 |
|  | Reverse: TGTTGCTACGCTTCTCAAACTG |  |
| *ZmGA20ox2* | Forward: CATCTCCATCGCCATGTTCCT | 106 |
|  | Reverse: GTAGTTGAACACCTTGTACCTGC |  |
| *ZmGA20ox7* | Forward: CCATGAGCAGCAGGAGTCGT | 93 |
|  | Reverse: CTGTCGGGCAGCCTGGTAAT |  |
| *ZmGA3ox1* | Forward: GGAGGAGTTCCACAAGCACA | 161 |
|  | Reverse: ACCTCGGGTACCAGTTGAGA |  |
| *ZmGA3ox3* | Forward: TGATGGCCACGTACAAGCAT | 154 |
|  | Reverse: GCTGATGATGACTGCTTGCG |  |
| Zea-actin | Forward: AATGGAACTGGAATGGTCAAGGC |  |
|  | Reverse: TGCCAGATCTTCTCCATGTCATCCCA |  |
