# Supplementary material for: Genome-wide analysis of gibberellin-dioxygenases gene family and their responses to GA applications in maize
Source: PLoS One. 2021 May 7;16(5):e0250349. doi: 10.1371/journal.pone.0250349 (PMC8104384; doi:10.1371/journal.pone.0250349)
Supplement: Attachment — Submitted filename: rebuttal letter(1)(1).docx [file pone.0250349.s004.docx]

Dear Editor:

On behalf of my co-authors, we thank you very much for giving us an opportunity to revise our manuscript, we appreciate editor and reviewers very much for their positive and constructive comments and suggestions. We have studied reviewer’s comments carefully and have made revision which marked in red in the paper. We have tried our best to revise our manuscript according to the comments. And the revised contents as follows:

1. Please ensure that your manuscript meets PLOS ONE's style requirements, including those for file naming. The PLOS ONE style templates can be found at

https://journals.plos.org/plosone/s/file?id=wjVg/PLOSOne_formatting_sample_main_body.pdf and

<https://journals.plos.org/plosone/s/file?id=ba62/PLOSOne_formatting_sample_title_authors_affiliations.pdf>

Thanks for your comments, we have confirmed that our manuscript meets PLOS ONE's style requirements.

2.  Thank you for stating the following in the Acknowledgments Section of your manuscript:

The work was carried out with the financial support of The National Key Research and
Development Program of China（2016YFD0101202）; Science and Technology Development Plan
of Jilin Province ( 20190301013NY).

i) We note that you have provided funding information that is not currently declared in your Funding Statement. However, funding information should not appear in the Acknowledgments section or other areas of your manuscript. We will only publish funding information present in the Funding Statement section of the online submission form.

Thanks for your comments, we have provided funding information in Funding Statement as follows: The work was carried out with the financial support of The National Key Research and Development Program of China（2016YFD0101202）; Science and Technology Development Plan of Jilin Province (20190301013NY).

ii) Please remove any funding-related text from the manuscript and let us know how you would like to update your Funding Statement. Currently, your Funding Statement reads as follows:

 "No. The funders had no role in study design, data collection and analysis, decision to publish, or preparation of the manuscript."

 iii) Please include your amended statements within your cover letter; we will change the online submission form on your behalf.

Thanks for your comments, the amended statements were added to the cover letter.

3. PLOS requires an ORCID iD for the corresponding author in Editorial Manager on papers submitted after December 6th, 2016. Please ensure that you have an ORCID iD and that it is validated in Editorial Manager. To do this, go to ‘Update my Information’ (in the upper left-hand corner of the main menu), and click on the Fetch/Validate link next to the ORCID field. This will take you to the ORCID site and allow you to create a new iD or authenticate a pre-existing iD in Editorial Manager. Please see the following video for instructions on linking an ORCID iD to your Editorial Manager account: <https://www.youtube.com/watch?v=_xcclfuvtxQ>

Thanks for your comments, we have established an ORCID iD and that it is validated in Editorial Manager.

4. Please include your tables as part of your main manuscript and remove the individual files. Please note that supplementary tables (should remain/ be uploaded) as separate "supporting information" files

Thanks for your comments, the tables have been included in our main manuscript.

[Note: HTML markup is below. Please do not edit.]

Reviewers' comments:

Reviewer's Responses to Questions

**Comments to the Author**

1. Is the manuscript technically sound, and do the data support the conclusions?

The manuscript must describe a technically sound piece of scientific research with data that supports the conclusions. Experiments must have been conducted rigorously, with appropriate controls, replication, and sample sizes. The conclusions must be drawn appropriately based on the data presented.

Reviewer #1: Yes

Reviewer #2: Yes

2. Has the statistical analysis been performed appropriately and rigorously?

Reviewer #1: Yes

Reviewer #2: N/A

3. Have the authors made all data underlying the findings in their manuscript fully available?

The [PLOS Data policy](http://www.plosone.org/static/policies.action#sharing) requires authors to make all data underlying the findings described in their manuscript fully available without restriction, with rare exception (please refer to the Data Availability Statement in the manuscript PDF file). The data should be provided as part of the manuscript or its supporting information, or deposited to a public repository. For example, in addition to summary statistics, the data points behind means, medians and variance measures should be available. If there are restrictions on publicly sharing data—e.g. participant privacy or use of data from a third party—those must be specified.

Reviewer #1: Yes

Reviewer #2: Yes

4. Is the manuscript presented in an intelligible fashion and written in standard English?

PLOS ONE does not copyedit accepted manuscripts, so the language in submitted articles must be clear, correct, and unambiguous. Any typographical or grammatical errors should be corrected at revision, so please note any specific errors here.

Reviewer #1: Yes

Reviewer #2: No

5. Review Comments to the Author

Please use the space provided to explain your answers to the questions above. You may also include additional comments for the author, including concerns about dual publication, research ethics, or publication ethics. (Please upload your review as an attachment if it exceeds 20,000 characters)

Reviewer #1: Ci et al. identified 27 gibberellin-dioxygenase proteins that are involved in GA biosynthesis and degradation in maize. The authors performed bioinformatic analysis and determined transcription levels of tested genes. Finally, five genes were speculated as potential genes for regulating GA concentrations, which may play essential roles in maize development. I only have minor comment to this study.
1. Abstract: line 14, “Gas” should be “GAs”. Please check the whole manuscript carefully and correct the mistake like this.

Thanks for your comments, we have carefully checked the whole manuscript carefully and correct the mistakes in the manuscript.
2. The names of all genes in the whole manuscript and figures should be italicized.

Thanks for your comments, the names of all genes in the whole manuscript and figures have been italicized.

3. Part 2.1: the method is not described in sufficient detail. How did the authors collect control samples? Did you collect control samples after water treatment (no GAs) for 6, 12, 24, 48, and72 h? If so, why is this data not shown in Figure 7? If you only collected control sample at 0 h (before GA treatment), when you collected GA-treated samples at 6, 12, 24, 48, and72 h, you found some gene expressions were changed, how to exclude the photoperiod and temperature effect on these gene expression?
4. In addition, in figure 7, “CK” is the abbreviation of cytokinin in english, please correct.

Thanks for your comments, we have changed it in the manuscript.
5. Part 2.6: line 7: H2O should be H2O; line 10, what is the meaning of “XX”?

Thanks for your comments, we have revised it in the manuscript.
6. The third paragraph of the discussion is not enough because many gibberellin-dioxygenase genes have been functional studied in many other plants, such as Arabidopsis and rice.

Thanks for your comments, we have changed it in the manuscript.
7. Reference: some journal are full name, but some are abbreviation.

Thanks for your comments, we have revised the references as the format of PLOS one.

Reviewer #2: In this manuscript, the authors reported Genome-wide identification of gibberellin-dioxygenases gene family in maize. By employing sequence-based bioinformatic analysis and qRT-PCR based gene expression determination, 27 Gibberellin-dioxygenases genes were identified in maize. The similar research had been done a lot in other plant species. From this prospective, this study in maize is lack of novelty. Still, it represents a standard report in relevant fields. I would like to suggest an acceptance after revision.

1.In the context of reported data in this study, the title of this manuscript is of over-presentation. Should the following be better: Genome-wide analysis of gibberellin-dioxygenases gene family and their responses to GA applications in maize

Thanks for your comments, the title have been changed to “Genome-wide analysis of gibberellin-dioxygenases gene family and their responses to GA applications in maize”.

2.Too much language flaws in tense, Singular/plural, and grammar. For instance in the abstract, Gibberellin-dioxygenases genes “plays”; In “the” present study; their “structures”; different expression “patterns”; tissue-specific, and so on. In the main text, for example, “In order to investigated”, wrong tense. Any way, it should be very easy to go through these by carefully self-reading. Even that, you may choose professional language editing services. IT MATTERS.
Thanks for your comments, we have chosen professional language editing service to revised the manuscript.
3. Some presentation are inexact, for instance:
(a)“ However, although much effort, the key genes and signaling pathways involved in GA remains elusive. “ The authors should define the context or put suitable limiting word in sentences like this one. In this case, at least the work in Arabidopsis, rice, and some others have accomplished a lot. Therefore, we know the key genes, the main pathways, and accordingly, we can refer to study their conserved counterparts in maize. So on so forth.
Thanks for your comments, we have changed it in the manuscript.
(b) “However, there is no systematic and complete investigation on gibberellin-dioxygenases genes family.” No, there have been quite a lot publications on different species. Should you please check and include as references? List below as examples:
Li, C., et al. (2019). "Comprehensive expression analysis of Arabidopsis GA2-oxidase genes and their functional insights." Plant Sci 285: 1-13.
Yan, J., et al. (2017). "Ectopic expression of GA 2-oxidase 6 from rapeseed (Brassica napus L.) causes dwarfism, late flowering and enhanced chlorophyll accumulation in Arabidopsis thaliana." Plant Physiol Biochem 111: 10-19.
Hu, Y.-X., Y.-B. Tao and Z.-F. Xu (2017). "Overexpression of Jatropha Gibberellin 2-oxidase 6 (JcGA2ox6) induces dwarfism and smaller leaves, flowers and fruits in Arabidopsis and Jatropha." Front Plant Sci 8: 2103.
Shan, X., Y. Li, et al. (2013). "Transcriptome Profile Analysis of Maize Seedlings in Response to High-salinity, Drought and Cold Stresses by Deep Sequencing." Plant Molecular Biology Reporter 31(6): 1485-1491.
Gou, J., S. H. Strauss, et al. (2010). "Gibberellins regulate lateral root formation in Populus through interactions with auxin and other hormones." Plant Cell 22(3): 623-639.
Yamaguchi, S. (2008). Gibberellin metabolism and its regulation. Annual Review of Plant Biology. Palo Alto, Annual Reviews. 59: 225-251.
Thanks for your comments, we have added these references in the manuscript.
(b)“the gibberellin-dioxygenases genes in response to GA are still poorly understood”, why? Too vague description.

Thanks for your comments, we have changed it in the manuscript.

6. PLOS authors have the option to publish the peer review history of their article ([what does this mean?](https://journals.plos.org/plosone/s/editorial-and-peer-review-process#loc-peer-review-history)). If published, this will include your full peer review and any attached files.


If you choose “no”, your identity will remain anonymous but your review may still be made public.


**Do you want your identity to be public for this peer review?** For information about this choice, including consent withdrawal, please see our [Privacy Policy](https://www.plos.org/privacy-policy).

Reviewer #1: No

Reviewer #2: No

[NOTE: If reviewer comments were submitted as an attachment file, they will be attached to this email and accessible via the submission site. Please log into your account, locate the manuscript record, and check for the action link "View Attachments". If this link does not appear, there are no attachment files.]

While revising your submission, please upload your figure files to the Preflight Analysis and Conversion Engine (PACE) digital diagnostic tool, <https://pacev2.apexcovantage.com/>. PACE helps ensure that figures meet PLOS requirements. To use PACE, you must first register as a user. Registration is free. Then, login and navigate to the UPLOAD tab, where you will find detailed instructions on how to use the tool. If you encounter any issues or have any questions when using PACE, please email PLOS at [figures@plos.org](mailto:figures@plos.org). Please note that Supporting Information files do not need this step.

*In compliance with data protection regulations, you may request that we remove your personal registration details at any time.* [*(Remove my information/details)*](https://www.editorialmanager.com/pone/login.asp?a=r)*. Please contact the publication office if you have any questions.*
